# Non-conventional interventions to prevent gonorrhea or syphilis among men who have sex with men: A scoping review

**DOI:** 10.3389/fmed.2022.952476

**Published:** 2022-09-20

**Authors:** Julien Tran, Christopher K. Fairley, Henry Bowesman, Ei T. Aung, Jason J. Ong, Eric P. F. Chow

**Affiliations:** ^1^Melbourne Sexual Health Centre, Alfred Health, Melbourne, VIC, Australia; ^2^Central Clinical School, Faculty of Medicine, Nursing and Health Sciences, Monash University, Melbourne, VIC, Australia; ^3^Centre for Epidemiology and Biostatistics, Melbourne School of Population and Global Health, The University of Melbourne, Melbourne, VIC, Australia

**Keywords:** gonorrhea, syphilis, MSM, sexually transmitted infection, review, intervention

## Abstract

**Objectives:**

We assessed nonconventional interventions that did not traditionally focus on increasing condom use and/or testing among men who have sex with men (MSM) and the evidence for these interventions.

**Methods:**

Guided by the Participants, Concept and Context (PCC) framework, we searched five online databases from inception to 9 August 2021 for original research on interventions that do not focus on increasing condom use and/or testing to prevent gonorrhea and/or syphilis in MSM. Two researchers screened titles and abstracts to assess eligibility, reviewed articles' full text and resolved discrepancies through discussion. We charted relevant study information, and the included studies were critically appraised.

**Results:**

Of 373 articles retrieved, 13 studies were included. These studies were conducted in Australia (*n* = 3), Belgium (*n* = 2), China (*n* = 3), the Netherlands (*n* = 1) and the US (*n* = 4). Two randomized controlled trials (RCTs) of doxycycline as pre-exposure prophylaxis (PrEP) and post-exposure prophylaxis (PEP) reduced any STI incidence (gonorrhea, syphilis, or chlamydia), but only doxycycline PEP significantly reduced syphilis incidence. Six studies of interventions that facilitated self-collection, self-examination, and self-testing, found varied evidence for gonorrhea and/or syphilis prevention. Four RCTs and one single-arm trial examined the efficacy of mouthwash, but the evidence remains inconclusive on whether mouthwash use can prevent transmission between men.

**Conclusion:**

We found evidence for doxycycline PEP in reducing syphilis incidence, evidence on the use of mouthwash to prevent gonorrhea transmission between men remains inconclusive. More evidence is needed for interventions that do not focus on increasing condom use and/or testing to prevent gonorrhea and/or syphilis.

## Introduction

Sexually transmissible infections (STIs), including chlamydia, gonorrhea, and syphilis, disproportionately affect men who have sex with men (MSM) (1–3). Globally, the World Health Organization has estimated that ~131 million people are infected with chlamydia each year, followed by 78 million people with gonorrhea and 6 million people with syphilis (4). While the incidence rate for chlamydia remains relatively stable (5–7), the incidence rates for both gonorrhea and syphilis have increased in high-income settings since the 2010s in MSM (1, 2) and heterosexuals (8–10). This review aimed to focus on the prevention of gonorrhea and/or syphilis among MSM.

Gonorrhea, caused by *Neisseria gonorrhoeae (N. gonorrhoeae)* (2, 11), can occur at the genitals (urethra/cervix), anorectum, and oropharynx. Since the 2010s, gonorrhea incidence among MSM attending sexual health clinics has significantly increased, particularly anorectal and oropharyngeal infections (12–16). The commonly accepted route for gonorrhea transmission between MSM is from an infected genital site to the anus and oropharynx through condomless sexual contact (17), but the importance of the oropharynx has recently been raised (18).

Due to its potential of becoming increasingly resistant to the antibiotics used for its treatment, gonorrhea has emerged as a global public health concern (19). Studies have demonstrated that due to lateral gene transfer, the oropharynx is an important anatomical site for antimicrobial resistance (AMR) (20–24). While decreasing gonorrhea incidence is key to reducing AMR (25), gonorrhea prevention strategies often focus on encouraging condom use. However, with the recent significant decline in condom use for anal sex (11, 26) and even less common use for oral sex among MSM (27), halting this decline or attempting to increase condom use to prevent gonorrhea can be challenging.

In addition to the decline in condom use, there have been concerns over the effectiveness of condoms based on research suggesting that the role of the penis may not be as important for transmission between men. For instance, research has shown that substantial bacterial loads of *N. gonorrhoeae* can be cultured in the saliva of individuals diagnosed with oropharyngeal gonorrhea (28, 29). Moreover, research has found that using saliva as a lubricant for anal sex is a risk factor for anorectal gonorrhea (30) and that tongue-kissing is an independent risk factor for oropharyngeal gonorrhea in MSM (31, 32). Given that most infections at the oropharynx are asymptomatic (33), interventions that target the oropharynx may be required for gonorrhea prevention.

Syphilis, caused by *Treponema pallidum* (*T. pallidum)*, continues to rise despite regular screening and contact tracing (34–38). Individuals with primary syphilis often present with lesions (or chancres) at the site of infection, and those with secondary syphilis present with relatively non-specific symptoms such as a skin rash. In contrast, individuals with early latent syphilis do not have symptoms (39). If left untreated, syphilis can lead to serious health concerns, including cardiac involvement (40), neurosyphilis (41), and ocular syphilis (41).

Previous research demonstrated that MSM who engaged in receptive anal sex only (where a partner's penis is inserted into their anus) were almost four times more likely to present with secondary syphilis than primary syphilis compared to men who did not engage in receptive anal sex (42). This suggests that a significant proportion of these men may have missed primary anorectal lesions and, therefore, progress from primary to secondary syphilis (43). Unrecognized oral and anal shedding of *T. pallidum* occurs most frequently in MSM with secondary syphilis. Therefore, progression toward this stage should be prevented to reduce the duration of infectiousness (43).

MSM who take PrEP are at a higher risk of acquiring syphilis, with an estimated incidence of 8.6 per 100 PY (39). Research has demonstrated that the recommended 3-monthly PrEP clinic appointments for syphilis screening have failed to detect a proportion of primary and secondary syphilis infections in MSM (39), suggesting that regular syphilis screening may be insufficient. Therefore, additional strategies are required to prevent onward transmissions of syphilis, especially in the context of higher syphilis incidence among HIV PrEP users (44).

There is an existing body of literature that consists of studies of interventions delivered by new media, such as websites, social media, or smartphone apps (45, 46) to increase condom use and clinic-based active recall interventions that aim to increase testing (where healthcare attendance is required) among MSM (38, 47). Therefore, the current review will not assess changes to condom use and/or testing in MSM, as an intervention outcome. Our scoping review aims to: (1) identify knowledge gaps and scope within the current body of literature on interventions that do not traditionally rely on increasing condom use and/or testing to prevent gonorrhea and/or syphilis in MSM and (2) synthesize knowledge to answer questions about the evidence for these interventions.

This scoping review focused on the following questions:

1. What non-conventional interventions that do not traditionally focus on increasing condom use and/or testing have been investigated or examined to prevent Gonorrhea and/or syphilis in MSM?

2. What is the evidence for these interventions?

As a guide to form our primary questions, we used the Participants, Concept and Context (PCC) framework, as recommended by Joanna Briggs Institute for scoping reviews (48). Here, we considered participants as MSM aged ≥16 years, including men living with HIV. Our concept focused on interventions that do not focus on condom use and/or testing to prevent gonorrhea and/or syphilis; and with regards to context, we focused on interventions in all possible settings or in different geographical regions and cultural contexts.

## Methods

We followed the methodology outlined in the manual for scoping reviews from the Joanna Briggs Institute (48).

### Eligibility criteria

We included original research evaluating interventions that do not focus on increasing condom use and/or testing to prevent gonorrhea and/or syphilis in MSM. We included biomedical interventions and self-managed interventions (i.e., self-collection, self-examination, and self-testing). Self-collection involves individuals only collecting their own samples, while self-testing required individuals to collect, test, and interpret sample results themselves. We also included interventions incorporating eHealth (i.e., health communication, new technology, technology-based engagement, and/or mHealth).

### Exclusion criteria

We excluded editorials, reviews, and studies of interventions that only focused on HIV and STIs that were not gonorrhea and/or syphilis, such as chlamydia, Hepatitis B, Hepatitis C, and *Mycoplasma genitalium*. We also excluded articles focusing on interventions focused on the use, promotion, or distribution of condoms, and campaigns that encourage testing without post-intervention evaluation data.

### Search strategy and study selection

**Figure 1:**
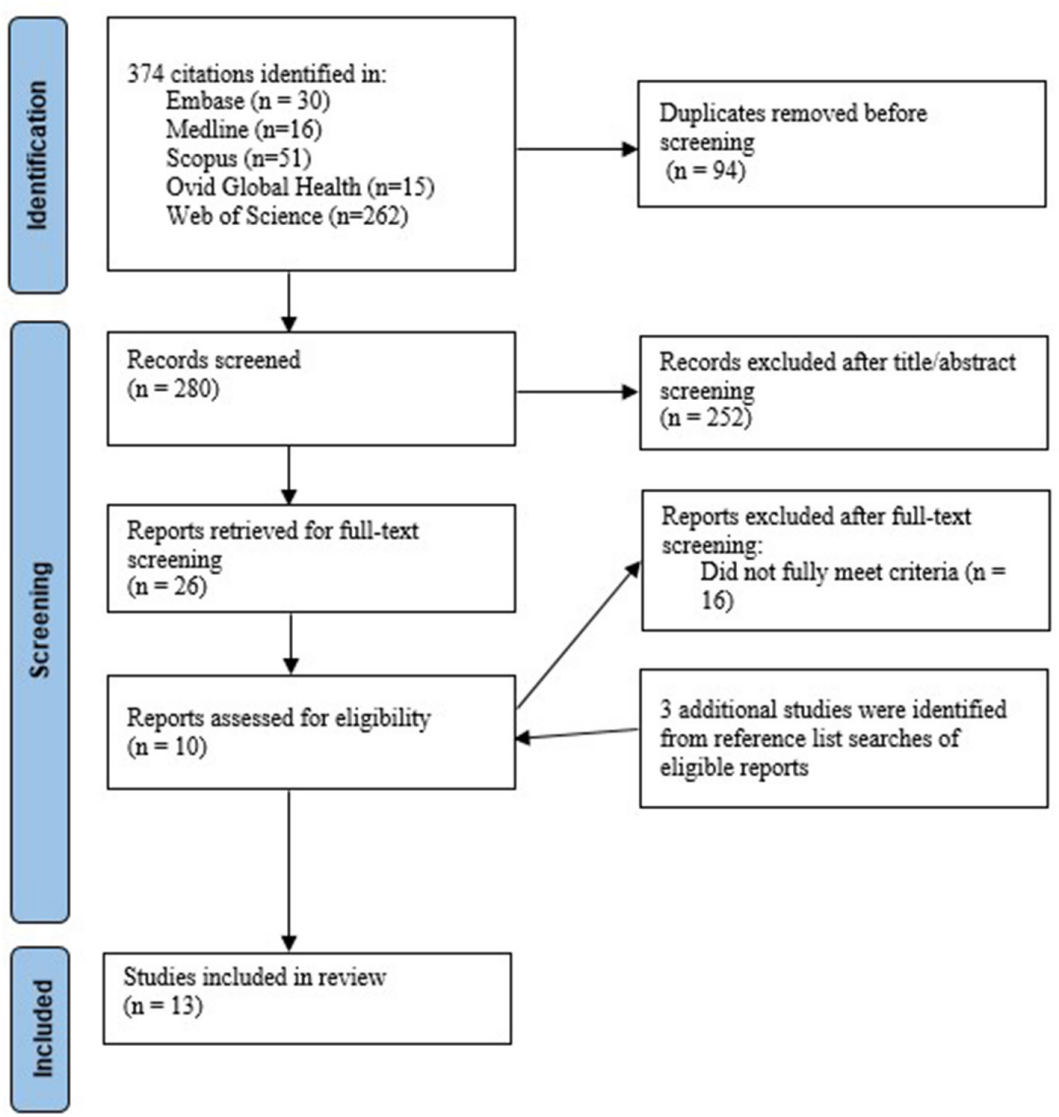
Flow chart of article selection.

We searched Ovid Medline, Ovid Global Health, Embase, Scopus and Web of Science Core Collection from inception to 9 August 2021. For each main concept, we used Medline to identify relevant medical subject headings (MeSH). The detailed search terms are provided in Supplementary Table S1. The results from our database searches were imported, and duplicates were removed in Endnote. Two researchers (JT and HB) independently screened the titles and abstracts of all the retrieved articles to assess eligibility, reviewed the full text of articles, and resolved discrepancies through discussion. Searches through the reference lists of included studies were conducted to find relevant studies (see Figure 1).

### Data charting and critical appraisal

For each study, we extracted information about the author, year of publication, study period, study location, study objective, study design (RCT, pre-and post-intervention, and post-intervention), study population, sample size, intervention description, and relevant findings. According to the study design, JT and ETA critically appraised the included studies against Joanna Briggs Institute checklists for RCTs (49), quasi-experimental studies and cross-sectional studies (50) for quality assurance when determining the evidence for the interventions.

## Results

We identified 374 articles from our database searches, of which 13 were included (10 from our database searches and three from our searches of reference lists of the included studies). Of 13 included studies, two investigated the efficacy of doxycycline prophylaxis, five examined the efficacy of mouthwash use, and six focused on the effectiveness of self-managed interventions in MSM. Of the six self-managed interventions, two required men to self-collect their samples only, two required men to self-examine their oral, anal, and urogenital areas, and two required men to self-test. Table 1 summarizes the characteristics of the 13 included studies.

**Table 1:** Characteristics of the included studies of non-conventional interventions for gonorrhea and/or syphilis prevention in MSM.

**References**	**Study period**	**Country**	**Study objective**	**Study design**	**Population**	**Sample size**
**Biomedical**
Bolan et al. (51)	Sep 2011–Jan 2012	USA	To determine whether daily doxycycline is efficacious in reducing STIs in high-risk groups	RCT 1:1 ratio 48-week follow-up	MSM and transgender women with syphilis history	30
Molina et al. (52)	Jul 2015–Jan 2016	France	To assess whether doxycycline as post-exposure prophylaxis can reduce STI incidence	RCT 1:1 ratio 10-month follow-up	MSM who had condomless sex and who used HIV PrEP	232
**Mouthwash**
Chow et al. (53)	May 2015–February 2016	Australia	To determine whether Listerine can inhibit *N. gonorrhoeae*	RCT 1:1 ratio No follow-up	MSM	196
Chow et al. (54)	Sep 2018–Feb 2020	Australia	To examine whether a 14-day course of mouthwash twice daily is efficacious in treating oropharyngeal gonorrhea	Parallel group, open-label RCT 1:1 ratio 28-day follow-up (Day 14: follow-up visit 1; Day 28: follow-up visit 2)	MSM	12
Chow et al. (55)	March 2016–October 2018	Australia	To compare the efficacy of Listerine Zero and Biotène mouthwashes in preventing gonorrhea in MSM.	RCT 1:1 ratio 12-week follow-up	MSM	530
Van Dijck et al. (56)	Apr 2019–Mar 2020	Belgium	To assess the use of an antiseptic mouthwash to prevent STIs	RCT 1:1 ratio 6-month follow-up (2 3-month visits)	MSM	343
Van Dijck et al. (57)	NS	Belgium	To assess efficacy of a mouthwash containing chlorhexidine in eradicating *N. gonorrhoeae* from the oropharynx	Single-arm pilot trial	MSM Asymptomatic oropharyngeal gonorrhea	3
**SELF-MANAGED**
**Self-collection**
Bardee et al. (58)	2016	USA	To evaluate the effectiveness of a novel STI self-collection program in HIV treatment clinic	Pre- and post-intervention study	MSM living with HIV	1,520 during the baseline year 1,510 during intervention year
Leenen et al. (59)	March-May 2018	The Netherlands	To pilot a free home-based STI self-collection program at an HIV treatment clinic	Post-intervention study	MSM living with HIV	28
**Self-examination**
Surie et al. (60)	2010–2011	USA	To increase self-examination to detect syphilis in MSM	Pre- and post-intervention	MSM	171 who had read the printed materials 735 who had not read the print materials
Taylor et al. (61)	February 2008–January 2009	USA	Evaluate intervention to increase oral and rectal self-examination for syphilis ulcers	Pre- and post- intervention study	Men living with HIV 76% MSM	689
Self-testing
Wu et al. (62)	Jun 2017–Nov 2019	China	To evaluate a program which involved social media-based secondary distribution of HIV/syphilis self-testing kits	Pre- and post-intervention study	MSM	371

### Quality assessment

Trial design was appropriate in all six RCTs included in this review. Of the included RCTs, true randomization occurred in all of six studies, while 67% concealed treatment group allocation, 67% blinded participants, 33% blinded those who delivered the treatment assignment, and 33% blinded outcome assessors to treatment assignment. Baseline data was similar in all of the six studies. There were no other differences in care or treatment received other than the intervention of interest across the compared groups in all of the six trials. Follow-up was complete in 67% of the studies and intention-to-treat analysis occurred in 50% of the studies. All studies measured their outcomes reliably and analyses were appropriately conducted, and all six studies measured their outcomes the same way in treatment groups. In the one single arm trial included, the temporal relationship of the cause and effect were clear. There was no control group, and therefore, group comparisons were not applicable. There were multiple measurements of the outcome, and these were measured in a reliable way and were appropriately analyzed.

All six studies checked against checklists for analytical cross-sectional studies clearly defined their inclusion criteria and described their subjects and setting in detail. All reliably measured their exposures. Of the six studies, 33% determined their sample using objective criteria and this criterion was not applicable to 33% of the studies. Identifying and adjusting for confounding was not applicable to all the studies. Most studies (83.3%) reliably measured and appropriately analyzed their outcomes.

### Biomedical interventions

Doxycycline is a tetracycline antibiotic used to treat bacterial infections. Two RCTs have demonstrated that using doxycycline as pre- and post-exposure prophylaxis may be an effective biomedical intervention to prevent STIs, including gonorrhea and/or syphilis, in MSM. The first was a US-based, 48-week open-label RCT of 30 MSM living with HIV and who also had previous syphilis infection randomized at a 1:1 ratio. This trial found that men who received 100 g of doxycycline pre-exposure prophylaxis daily were significantly less likely to test positive for any of the selected bacterial STIs (with 73% reduction in syphilis, gonorrhea, chlamydia or a combination of these STIs) compared to men who received contingency management, where there was a financial incentive if they remained STI-free throughout the trial (*p* = 0.02) (51). When incidence for gonorrhea, chlamydia and syphilis was examined individually, there were no significant differences in incidence between the men in both groups. There were no significant differences in sexual risk behaviors between men in both groups.

In France, a 10-month open-label RCT of 232 MSM and transgender women randomized at a 1:1 ratio, found that there was a 47% relative reduction in the risk of acquiring any STIs (syphilis, gonorrhea, chlamydia, or a combination of these STIs) in individuals who received 200 g of doxycycline as post-exposure prophylaxis (PEP) within 24–72 h after sex compared to individuals who did not receive doxycycline PEP. There was a 73% relative risk reduction in acquiring syphilis in individuals who received doxycycline PEP compared to those who did not receive doxycycline PEP (*p* = 0.047). However, the relative risk of acquiring gonorrhea did not significantly differ between individuals in both groups (52). These RCTs demonstrated that results for specific STIs varied depending on whether doxycycline was used as pre-exposure prophylaxis or post-exposure prophylaxis.

### Self-managed behavioral interventions

#### Self-collection

Self-collection, where men only collect their own pharyngeal, rectal and urine specimens, has been found to increase detection among MSM. For instance, in a US study by Barbee et al. (58), men were given kits to self-collect their samples by following instructional posters placed on the walls of a room designated for self-collection at their local sexual health clinic during the intervention year. Baseline data on infections was collected the year prior to the intervention year. Self-collection during the intervention year detected 147 gonorrhea infections, which was 49 (31 oropharyngeal; 18 anorectal) more infections compared to the 98 infections detected during the baseline year, resulting in a 50% increase in detection. Sexual practices were not measured.

Self-collection at home can also increase STI detection among MSM. Conducted in the Netherlands, a study of 28 MSM living with HIV who were offered free home-based kits for self-collection of pharyngeal, rectal and urine specimens, and blood samples for syphilis testing at their routine care visit by healthcare professionals, found that 17.9% (5/28) were newly diagnosed with one or more STIs (59).

#### Self-examination

Several studies have been investigated self-examination, which requires men to examine their oral, anal, and urogenital areas, as an intervention to prevent syphilis in MSM. A US-based study involved 689 men (76% MSM) living with HIV who received posters of primary and secondary syphilis lesions before their quarterly clinic visits. Syphilis prevention messages were included at the top of each poster, for example: “Sores caused by syphilis are painless and can be found in the mouth, anus, rectum, and penis”, or “Neurosyphilis can cause blindness, hearing loss, cognitive decline, stroke, and chronic headaches”. At baseline and at each of their quarterly visits, the men were asked questions about unprotected oral and anal sex with their regular partner, or casual or anonymous partners, and whether they had self-examined their oral and anal areas for syphilis lesions. There were no significant differences in the men's number of unprotected oral and anal sex activities with regular, casual, or anonymous partners at baseline through to their third clinic visit. However, self-examination of oral and anal areas increased from 46% at baseline to 72% among men with three clinic visits (*p* < 0.001) (61).

Another US-based study involved 906 MSM who were provided with brochures about syphilis symptoms, transmission, and prevention after their clinic visits. The men were asked whether they had read the brochures and those who responded “yes” were grouped as having read the brochures and those who responded “no” were grouped as not having read the brochures. The study found that men who read the brochures from a previous visit (*n* = 171/906) were significantly more likely than men who did not read the brochures (*n* = 735/906) to self-examine their oral (adjusted prevalence ratio; aPR = 1.2, 95% CI: 1.14–1.36, *p* < 0.05), anal (aPR = 1.3, 95% CI 1.15–1.52. *p* < 0.05), genital areas (aPR = 1.1, 95% CI: 1.01–1.14, *p* < 0.05) and their skin (aPR = 1.2, 95% CI 1.05–1.19) for at least once a week and were more likely to examine their partners' oral (aPR = 1.6, 95% CI 1.10–1.2.26, *p* < 0.05) and anal areas (aPR = 1.3, 95% CI 1.03–1.73, *p* < 0.05) for at least once a week (60). There were no significant differences in examining partner's genitals and skin between men in both groups.

#### Self-testing

Self-testing involves individuals collecting and testing their specimens and interpreting the results. One way to increase self-testing is through secondary distribution, which involves giving an individual multiple self-testing kits to distribute to people within their social networks. A study conducted in Zhuhai, China, recruited 331 MSM (“indexes”) who distributed HIV/syphilis self-tests to 281 individuals within their social networks (“alters”) (62). The self-tests had to be ordered through WeChat (a multifunctional social app) and were mailed out to the 331 men. Using Quick Response (QR) codes, pictures of test results were anonymously uploaded to WeChat. However, the study concluded that there were no significant differences in the reactive syphilis results between the indexes and alters.

A study by Yang et al. (63), also conducted in Zhuhai, China, assessed HIV/syphilis self-testing among social networks of sexual health influencers and non-influencers. Men were sexual health influencers if they scored >3 and sexual health non-influencers if they scored <3 on six items using a 5-point Likert-type scale. The six items assessed whether men could influence others to seek advice about HIV/STI issues and how often they discussed HIV/STI topics with other people. The study found that sexual health influencers were more likely to influence people within their social networks to upload their test results using QR codes to WeChat compared to sexual health non-influencers (adjusted rate ratio = 2.07, 95% CI: 1.59–2.69). Compared to the alters of sexual health non-influencers, sexual health influencers had more alters who were from a rural area (45.5 vs. 23.8%, *p* < 0.001), did not attend university (57.7 vs. 37.1%, *p* < 0.001), and who had multiple casual sex partners (25.2 vs. 11.9%, *p* < 0.001) in the previous 6 months (63).

### Mouthwash as an intervention

In the late 2010s, mouthwash was proposed as an intervention for gonorrhea prevention and treatment by several researchers. We identified three RCTs examining the efficacy of mouthwash in preventing STIs; one RCT in Belgium (56) and two were conducted in Australia (53, 55). Additionally, two RCTs examined the efficacy of using mouthwash as treatment for oropharyngeal gonorrhea (54, 57).

The randomized, placebo-controlled, crossover trial conducted in Belgium investigated the efficacy of daily use of Listerine mouthwash and mouthwash use before and after sex among 343 MSM taking PrEP and who also had an STI in the previous 24 months. This trial found men who used Listerine did not significantly reduce STI incidence (incidence rate ratio 1.17, 95% CI 0.84–1.64) compared to men who used the placebo mouthwash. In the Listerine-placebo group, the STI incidence was 140.4 per 100 PY during the Listerine phase and 102.6 per 100 PY during the placebo phase. In the placebo-Listerine group, the STI incidence rate was 133.9 per 100 PY during the placebo phase and 147.5 per 100 PY during the Listerine phase (56). A significantly higher proportion of oropharyngeal gonorrhea cases were detected when using Listerine than when using placebo (OR 5.78, 95% CI 1.52–136.56, *p* = 0.024). However, Listerine use was not significantly associated with gonorrhea cases at any anatomical site (OR 1.48, 95% CI 0.81–2.83). There were no significant differences in syphilis cases between Listerine use and placebo.

The first ever RCT on mouthwash was conducted in Australia that involved 196 MSM with untreated oropharyngeal gonorrhea. Men were randomized at 1:1 ratio to either using Listerine Cool Mint mouthwash (containing 21.6% alcohol) or a saline solution. Men were asked to rinse and gargle 20 ml of the allocated solution for 1 min. Swabs at the tonsillar fossae and posterior oropharynx were taken before and 5 min after the men rinsed and gargled. This trial found that culture positivity on the pharyngeal surface was significantly lower in men who use Listerine mouthwash (52%) compared to men who used the saline solution (84%) (*p* = 0.013) (53).

The second mouthwash RCT was the OMEGA trial and involved 530 MSM in Australia. Men were randomized at 1:1 ratio to either using Listerine Zero (0% of alcohol) mouthwash or Biotène mouthwash (i.e., a mouthwash did not have any inhibitory effect against *N. gonorrhoeae*). Men were asked to rinse and gargle the allocated mouthwash for 60 s at least once daily over 12 weeks. This trial found that the cumulative incidence of oropharyngeal gonorrhea did not significantly differ between men in the Listerine mouthwash group and men in the Biotène mouthwash group (adjusted risk difference 3.1%, 95% CI −1.4 to 7.7) (55). However, the trial also found that a significant reduction in urethral gonorrhea (<1 vs. 4%; adjusted risk difference −4.3%, 95% CI −7.4 to −1.3) between men in the Listerine Zero group compared to the Biotène mouthwash group, but not for anorectal gonorrhea (7 vs. 4%; adjusted risk difference 2.5%, 95% CI −1.9 to 7.0). There were no significant differences in syphilis incidence between the men in both groups (adjusted risk difference −0.4%, 95% CI −2.2 to 1.3).

While the first two RCTS from Australia investigated the efficacy of mouthwash for STI prevention, the third RCT in Australia investigated mouthwash as potential STI treatment. The OMEGA2 trial was an RCT of 12 Australian MSM with untreated oropharyngeal gonorrhea who were randomized at 1:1 ratio to either receive a 14-day course of mouthwash twice a day or standard antibiotic treatment to cure their oropharyngeal gonorrhea (54). Men were asked to abstain from sex and kissing for 14 days after enrolling in the study. Of those who returned on day 14, the cure rate for oropharyngeal gonorrhea was 20% (1/5) for those randomly assigned to the mouthwash group, while the cure rate was 100% (6/6) for the standard treatment group (54). This trial failed to demonstrate using mouthwash as an alternative treatment for oropharyngeal gonorrhea and therefore, the trial was terminated early.

An open-label single-arm trial which also investigated mouthwash as treatment for STIs was conducted in Belgium and involved in 6 MSM with asymptomatic oropharyngeal gonorrhea. The men were required to gargle mouthwash (containing 0.2% mg/mL chlorhexidine) twice daily over 6 days. Three men exited the trial before their day 7 visit. The use of mouthwash containing chlorhexidine failed to eradicate *N. gonorrhoeae* from the oropharynx of three asymptomatic men (efficacy 0%; 95% confidence interval, 0–56.1%). Therefore, this trial was terminated early.

## Discussion

We identified studies of non-conventional interventions to prevent gonorrhea and/or syphilis in MSM conducted in different geographical regions and cultural contexts. While these interventions seemed to be highly acceptable to the men, there are potential issues related to terminology, transferability, and sustainability of these interventions that need to be considered if future interventions that do not focus on increasing condom use and/or testing are going to target high-risk, hard-to-reach groups and to be implemented at the population level.

The efficacy for doxycycline prophylaxis has only been demonstrated in clinical trials. Doxycycline pre-exposure prophylaxis did not reduce syphilis incidence in one study, which was most likely due to its small sample size (i.e., 15 patients per arm) (51), but doxycycline post-exposure prophylaxis significantly reduced syphilis incidence in MSM (52). Given the significance of antibiotic resistance, it is important to establish the effectiveness of doxycycline PEP so that this benefit can be evaluated within the context of the substantial increase in the use of antibiotics. An RCT “Syphilaxis” examining the efficacy of doxycycline PrEP in reducing the incidence of STIs (including gonorrhea, chlamydia, and syphilis) among MSM is underway in Australia (Identifier: NCT03709459). Additionally, this trial will also evaluate resistance in the gut microbiota among men using doxycycline PrEP. Four other RCTs are in progress or development for doxycycline prophylaxis to prevent STIs in MSM (64).

We found that there are some inconsistencies and misuse of the terminology related to self-testing. For instance, self-testing requires individuals to collect and test their own specimen and interpret the results themselves. Still, some interventions that only required individuals to self-collect their samples were labeled as self-testing. There is some evidence for interventions that use instructional materials to influence self-collection behaviors and in turn, detect gonorrhea (58). Similarly, there is also evidence for using educational materials such as syphilis prevention brochures to increase self-examination and partner-examination (60, 61), however, without gonorrhea and syphilis infections reported as associated outcomes, the evidence is insufficient.

Secondary distribution as an approach to increase the number of new self-testers among people within already established social networks holds some promise, particularly distribution by sexual health influencers (63). This approach can be adapted to other geographical regions and cultural contexts and cater to the needs of high-risk, hard-to-reach groups, such as men residing in more isolated rural areas with a high number of male casual sex partners (62). There are two RCTs underway in China; one examining the efficacy of social network distribution of syphilis self-testing in MSM (Identifier: ChiCTR2000036988) (65) and the other examining the efficacy of free syphilis self-tests in MSM (Identifier: ChiCTR1900022409) (66). The findings from these studies may help determine the transferability of these interventions.

Our review found RCTs that assessed the efficacy of mouthwash use to prevent oropharyngeal gonorrhea in MSM. One RCT demonstrated that culture positivity on the pharyngeal surface was significantly lower in men who gargled mouthwash compared to those who gargled the saline solution (53), suggesting that mouthwash use can increase gonococcal clearance. However, this was an immediate effect 5 min after the use of mouthwash and the effectiveness of consistent use or long-term use of mouthwash is unclear. Two further RCTs examining the efficacy of daily use of Listerine mouthwash for gonorrhea prevention but the results are inconclusive (55, 56). Additionally, two other RCTs also revealed that mouthwash appears to be an ineffective treatment for oropharyngeal gonorrhea compared to antibiotics (54, 55). The oropharynx plays an important role in gonorrhea transmission; and therefore, more conclusive evidence is needed to inform preventive options that target the oropharynx at the population level.

Mathematical modeling has indicated that if upon presentation for STI testing, 30% of MSM are vaccinated with a gonococcal vaccine with 50 or 100% efficacy, gonorrhea prevalence could be reduced by 94 or 62%, respectively, within 2 years (67). There is some evidence for the cross-over protection from an outer membrane vesicle Neisseria *meningitidis* serogroup B (MeNZB) vaccine against *N. gonorrhoeae*. For instance, a retrospective case-control cohort study of 14,730 sexual health clinic attendees, found that gonorrhea incidence decreased by 31% among vaccinated individuals (68). Currently, a 24-month multi-center, double-blinded, RCT is underway to investigate the efficacy of the 4CMenB vaccine to reduced gonorrhea in MSM (Identifier: NCT04415424).

This scoping review has several limitations. First, this review may not have been able to identify and in turn, may have missed published studies of self-managed behavioral interventions that were labeled using terms other than self-screening, self-testing, self-examination, and/or self-collection. Furthermore, while the studies we assessed were conducted in various geographical regions and cultural contexts, the potential for transferability of the findings is yet to be determined due to the lack of conclusive evidence.

## Conclusions

While there is some promise in several of the alternative strategies assessed, more robust evidence is needed to support their effectiveness and transferability. Recent evidence supports the effectiveness of doxycycline prophylaxis (69), but there are concerns about the development of AMR and whether the benefit outweighs the potential overuse of doxycycline and the risk of AMR. Questions have also been raised about the cost-effectiveness and sustainability of doxycycline prophylaxis. While it is currently unavailable, an effective vaccine for preventing gonorrhea in MSM and other groups who are at risk could reduce infections markedly. Several trials investigating the efficacy of the 4CMenB vaccine for gonorrhea prevention are currently underway. While the use of antibacterial mouthwash can inhibit the growth of *N. gonorrhoeae* in the oropharynx, there is no evidence that daily use of antibacterial mouthwash could prevent individuals from acquiring gonorrhea. There is no evidence for self-managed strategies, such as regular anorectal self-examination and syphilis self-testing facilitated by social media platforms, like WeChat, for syphilis control in MSM, as these strategies are currently not supported by sufficient data linked to changes in men's syphilis infections. However, investigations are currently underway to examine the effectiveness of these self-managed strategies for syphilis control among MSM.

## Data availability statement

The original contributions presented in the study are included in the article/Supplementary material, further inquiries can be directed to the corresponding author/s.

## Author contributions

EC, JO, and JT conceived and designed the review. JT performed the search, screened, charted information for eligible studies, and wrote the first draft of the manuscript. HB assisted with screening for eligible studies. EA assisted with the critical appraisal. All authors were involved in the interpretation of findings, commented on manuscript drafts, and contributed to the final version of the manuscript.

## Funding

EC and JO are supported by an Australian National Health and Medical Research Council (NHMRC) Emerging Leadership Investigator Grant (GNT1172873 for EC and GNT1104781 for JO). CF was supported by an Australian NHMRC Leadership Investigator Grant (GNT1172900). JT and EA was supported by the Australian Government Research Training Program (RTP) Scholarship.

## Conflict of interest

The authors declare that the research was conducted in the absence of any commercial or financial relationships that could be construed as a potential conflict of interest.

## Publisher's note

All claims expressed in this article are solely those of the authors and do not necessarily represent those of their affiliated organizations, or those of the publisher, the editors and the reviewers. Any product that may be evaluated in this article, or claim that may be made by its manufacturer, is not guaranteed or endorsed by the publisher.
