# Supplementary material for: Non-conventional interventions to prevent gonorrhea or syphilis among men who have sex with men: A scoping review
Source: Front Med (Lausanne). 2022 Sep 20;9:952476. doi: 10.3389/fmed.2022.952476 (PMC9530550; doi:10.3389/fmed.2022.952476)
Supplement: Supplementary file 1 [file Table_1.docx]

**Supplementary Table S1.** Search strategy (titles and abstracts)

| **Number** | **Search terms** |
| --- | --- |
| **1** | MSM or men who have sex with men |
| **2** | Gay |
| **3** | Bisexual* |
| **4** | homosexual* |
| **5** | 1 OR 2 OR 3 OR 4 |
| **6** | eHealth |
| **7** | Mouthwash |
| **8** | Antibiotic |
| **9** | Doxycycline |
| **10** | Self-screen* |
| **11** | Self-test* |
| **12** | Self-examine* |
| **13** | Self-collect* |
| **14** | 6 OR 7 OR 8 OR 9 OR 10 OR 11 OR 12 OR 13 |
| **15** | Neisseria Gonorrhoea |
| **16** | Gonococc* |
| **17** | Gonorrhoea |
| **18** | Treponema pallidum |
| **19** | Syphilis |
| **20** | 15 OR 16 OR 17 OR 18 OR 19 |
| **21** | 5 AND 14 AND 20 |
